# The Effectiveness of a Mobile Phone–Based Physical Activity Program for Treating Depression, Stress, Psychological Well-Being, and Quality of Life Among Adults: Quantitative Study

**DOI:** 10.2196/46286

**Published:** 2023-06-26

**Authors:** Hyungsook Kim, Kikwang Lee, Ye Hoon Lee, Yoonjung Park, Yonghyun Park, Yeonwoo Yu, Jaeyoung Park, Sihyeon Noh

**Affiliations:** 1 Hanyang Digital Healthcare Center Hanyang University Seoul Republic of Korea; 2 Department of Cognitive Sciences School of Intelligence Hanyang University Seoul Republic of Korea; 3 Graduate School of Public Policy Hanyang University Seoul Republic of Korea; 4 Department of Sport, Health, and Rehabilitation College of Physical Education Kookmin University Seoul Republic of Korea; 5 Division of Global Sport Industry Hankuk University of Foreign Studies Gyeonggi-do Republic of Korea; 6 Department of Health & Human Performance University of Houston Houston, TX United States

**Keywords:** depressive symptoms, mobile intervention, exercise, internet-based fitness, mental health

## Abstract

**Background:**

Depression is a substantial global health problem, affecting >300 million people and resulting in 12.7% of all deaths. Depression causes various physical and cognitive problems, leading to a 5-year to 10-year decrease in life expectancy compared with the general population. Physical activity is known to be an effective, evidence-based treatment for depression. However, people generally have difficulties with participating in physical activity owing to limitations in time and accessibility.

**Objective:**

To address this issue, this study aimed to contribute to the development of alternative and innovative intervention methods for depression and stress management in adults. More specifically, we attempted to investigate the effectiveness of a mobile phone–based physical activity program on depression, perceived stress, psychological well-being, and quality of life among adults in South Korea.

**Methods:**

Participants were recruited and randomly assigned to the mobile phone intervention or waitlist group. Self-report questionnaires were used to assess variables before and after treatment. The treatment group used the program around 3 times per week at home for 4 weeks, with each session lasting about 30 minutes. To evaluate the program’s impact, a 2 (condition) × 2 (time) repeated-measures ANOVA was conducted, considering pretreatment and posttreatment measures along with group as independent variables. For a more detailed analysis, paired-samples 2-tailed *t* tests were used to compare pretreatment and posttreatment measurements within each group. Independent-samples 2-tailed *t* tests were conducted to assess intergroup differences in pretreatment measurements.

**Results:**

The study included a total of 68 adults aged between 18 and 65 years, who were recruited both through web-based and offline methods. Of these 68 individuals, 41 (60%) were randomly assigned to the treatment group and 27 (40%) to the waitlist group. The attrition rate was 10.2% after 4 weeks. The findings indicated that there is a significant main effect of time (*F*_1,60_=15.63; *P*=.003; *η*_p_^2^=0.21) in participants’ depression scores, indicating that there were changes in depression level across time. No significant changes were observed in perceived stress (*P*=.25), psychological well-being (*P*=.35), or quality of life (*P*=.07). Furthermore, depression scores significantly decreased in the treatment group (from 7.08 to 4.64; *P*=.03; Cohen *d*=0.50) but not in the waitlist group (from 6.72 to 5.08; *P*=.20; Cohen *d*=0.36). Perceived stress score of the treatment group also significantly decreased (from 2.95 to 2.72; *P*=.04; Cohen *d*=0.46) but not in the waitlist group (from 2.82 to 2.74; *P*=.55; Cohen *d*=0.15).

**Conclusions:**

This study provided experimental evidence that mobile phone–based physical activity program affects depression significantly. By exploring the potential of mobile phone–based physical activity programs as a treatment option, this study sought to improve accessibility and encourage participation in physical activity, ultimately promoting better mental health outcomes for individuals with depression and stress.

## Introduction

### Background

Depression is a mental health problem that affects >300 million people worldwide, accounting for 12.7% of all deaths [1]. Depression causes various types of physical and cognitive problems, such as the loss of appetite, sleep disturbances, helplessness, and suicidal thoughts and attempts [2]. Therefore, it has been reported that the mean life expectancy of people with depression is 5 to 10 years shorter than that of the general population [3]. Similar to the global trend, the number of people with depression in South Korea is increasing every year, and medical expenses are also gradually increasing. As the COVID-19 pandemic and social distancing continued in South Korea, 18.9% of the population was classified as a risk group for depression, and the number of people with depression has increased by >30% from approximately 750,000 in 2018 to 830,000 in 2020 [4]. Furthermore, according to the 2018 National Health and Nutrition Examination Survey [5], the stress awareness rate among adults in Korea was 29.1%, and it persisted at approximately 30% over the following 3 years. This means that 3 out of 10 adults feel *very much* or *a lot of* stress in their daily lives. Therefore, interest in intervention methods for managing depression in adults is increasing [6].

The traditional methods of preventing and treating depression include drug therapy, psychotherapy (eg, cognitive therapy and interpersonal therapy), and combinations of both [7]. Antidepressants—for example, selective serotonin reuptake inhibitors—are often used as the first-line treatment for depression because of their advantages of scalability and accessibility [8]. Despite the demonstrable biological underpinnings of the antidepressant mechanisms supporting the use of these drugs [7], it has been reported that only approximately 50% of individuals who take such medication achieve a statistically significant reduction in depressive symptoms [9]. In addition, previous literature has demonstrated the side effects and increased mortality associated with the difficulty of maintaining drug compliance, increased weight and diabetes risk, sexual dysfunction, and various diseases. Moreover, if antidepressants are stopped before recovery, symptoms seem to worsen rapidly, and it is difficult to continue drug treatment because of negative perceptions about drug consumption and dependency [10]. Thus, there is a need for alternative treatment methods that can overcome these issues.

Cognitive behavioral therapy is an alternative treatment that is widely recognized as one of the most effective evidence-based therapies for depression. Studies conducted worldwide have reported small to medium effects on mitigating depression [11]. However, despite the availability of effective treatments, a large proportion of people with depression do not seek or receive professional help, with studies reporting that up to 80% of adolescents with depression do not receive adequate treatment [12]. Barriers to treatment are reported to include prejudice, accessibility issues, and cost issues associated with psychiatric treatment [13]. These issues are not limited to specific regions or countries, as people worldwide often experience reluctance to seek treatment owing to the fear of stigma and high cost of therapy. Furthermore, cognitive behavioral therapy is often a weekly treatment for at least 10 sessions, which can be financially burdensome for many individuals. Therefore, alternative treatment methods that address or avoid these difficulties are needed [14].

Physical activity and exercise can be an effective alternative form of depression treatment [15]. They exert antidepressant effect through multiple biological and psychosocial pathways [16-18]. Kandola et al [7] conducted a review to explore the relationship between physical activity and depression, aiming to understand the mechanisms by which physical activity exerts its antidepressant effect. The review highlights the interdependent changes that occur in the brain owing to exercise, creating a protective environment against depression. These changes include increased levels of neurotrophins, improved brain structure, and enhanced functioning in areas implicated in depression and stress regulation. Exercise produces changes in the brain through various pathways, including neuroplasticity, which is disrupted in depression [19]. Studies have found that exercise can increase the volume of the hippocampus and cortical regions, and high levels of cardiorespiratory fitness are associated with large volumes in these areas [20-22]. In addition, exercise interventions can reduce chronic low-grade inflammation, decrease depressive symptoms, and mitigate oxidative stress, which can contribute to depression. Regular exercise may also help to dampen hypothalamic-pituitary-adrenal axis activity and cortisol sensitivity, leading to increased resilience to stress, and it may have a direct influence on the neuroendocrine system, helping to reduce cortisol levels in people with depression [23]. The review concludes that psychosocial factors, such as self-esteem and self-efficacy, also interact with biological changes to influence depression. Therefore, it is important to note that the mechanisms by which physical activity exerts its antidepressant effect are multifaceted and may vary depending on the individual. Several meta-analyses have found that exercise can reduce the symptoms of depression with a moderate to large effect size and that exercise can be a useful addition to pharmacotherapy and psychotherapy [24-30].

Recently, the COVID-19 pandemic has increased the need for mental health and well-being management in non–face-to-face environments [31]. Depression is strongly associated with social phobias [32], which limit people’s ability to exercise in spaces with other people, such as fitness centers. Therefore, easy-to-access and easy-to-use internet-based or mobile phone–based physical activity apps can be good alternatives for people with depression. Mobile phone–based physical activity programs have the potential to provide significant benefits as an adjunct to professional treatment for depression. These benefits include increased convenience, adherence, personalization, social support, and cost-effectiveness [33]. By enabling individuals to engage in physical activity regularly, mobile phone–based programs with reminder and tracking features can increase adherence to physical activity interventions [34]. Furthermore, these programs can be tailored to an individual’s specific needs and preferences, potentially increasing motivation and engagement. Social support features, such as web-based communities or peer support, are also common in mobile phone–based programs and may help individuals to stay motivated and engaged in the program [35]. Finally, mobile phone–based programs may be more cost-effective than traditional in-person interventions, making them more accessible to individuals who may not have the financial resources for traditional treatment [36]. Numerous studies have demonstrated the effectiveness of mobile phone–based physical activity programs at home for adults in treating depression [37-40].

In light of the increasing demand for non–face-to-face mental health management during the COVID-19 pandemic, there has been a surge in the development and evaluation of technology-based physical activity interventions [36]. However, despite their potential benefits, there is a lack of studies exploring the effectiveness of web-based physical activity interventions in reducing depression and improving mental health outcomes such as perceived stress [41], psychological well-being [42], and quality of life [36,43]. Moreover, the existing studies have produced mixed outcomes, and many have not included control groups [36], highlighting the need for further studies to examine the mental health outcomes of web-based physical activity interventions.

### Objective

To address this issue, we investigated the effectiveness of a mobile phone–based physical activity program among adults in South Korea by examining various mental health indicators such as depression, perceived stress, psychological well-being, and quality of life. Specifically, we hypothesized that the mobile phone–based physical activity program will lead to significant improvement in depression, perceived stress, psychological well-being, and quality of life in the intervention group compared to the control group. We hope that this study of mobile phone–based physical activity programs, which can be easily accessed by people with depression at home, will contribute to mitigating the negative impact of mental health issues. It is our aspiration that this study will provide more comprehensive guidelines for decision makers to promote and execute mobile phone–based exercise interventions for individuals with depression and stress.

## Methods

### Participants

The participants were recruited through various channels, including the Hanyang Digital Healthcare Center website [44], the Hanyang Happiness Dream Counseling Center, and the related blog [45]. In addition, recruitment efforts included the distribution of posters, flyers, and local newsletters in the community. Participants were recruited from the first week of March 2022 to the third week of April 2022 through the web-based and offline advertisements. Participants completed the baseline questionnaire in the fourth week of April 2022 and the posttest questionnaire in the first week of June 2022. The study enrolled adults who were aged between 18 and 65 years, were fluent in Korean, had basic knowledge about the internet, and could attend the 3 in-person appointments at the project locations (ie, Hanyang Digital Healthcare Center). To be eligible, they needed to have access to the internet and pass the Physical Activity Readiness Questionnaire.

### Sample Size Calculation

To calculate the sample size, the G*power 3 program was used, with power set to 95% and significance level of .01 for precise testing. The study measured the degree of depression relief in the fourth week using the sixth week’s measurement from a previous study. The effect size was determined to be 1.1957131, and the minimum number of study participants required was calculated to be 54, with 27 participants per group. Given that the study was on the general public who felt depressed for 8 weeks, a dropout rate of 20% was set, and a total of 65 study participants were recruited.

### Procedure

Following the randomization process, participants in the intervention group were shown the mobile phone–based physical activity program during their initial appointment and were provided with instructions about how to access and use it at home. To ensure that participants in the treatment group completed the exercise program as intended, we provided them with a set of instructions and guidelines and a calendar to track their progress. We also asked them to complete a Google Form after each exercise session to confirm that they had completed the exercise and to report any issues or concerns. By using the Google Form to collect data about participant compliance, this study minimized any potential bias that could arise from direct contact with participants. Participants in the study were compensated for their time and effort with a monetary reward of KRW ₩100,000 (US $75.76) for the treatment group and KRW ₩50,000 (US $37.88) for the control group at the end of the study. Participants were informed about the compensation at the beginning of the study and were reminded about the amount and timing of payment before the last session. The monetary reward was intended to incentivize participation and improve retention rates.

### Ethics Approval and Informed Consent

This study was approved by the Hanyang University (the first authors’ institution) institutional review board (HYUIRB-202203-010-2). We obtained the necessary approvals before starting the study to ensure that ethical standards were met, and the rights of the participants were protected. The purpose and procedure of the study were explained before starting of the study, and only those who provided written informed consent and voluntarily participated were included.

### Instrument

#### Overview

The Korean versions of the Patient Health Questionnaire–9 (PHQ-9), Perceived Stress Scale, and World Health Organization (WHO)–5 Well-Being Index were used in this study. These scales have been previously validated in the literature [46]. Translation and cultural adaptation were conducted using established guidelines, including forward and backward translations and cultural adaptation by a team of bilingual experts [47]. The quality-of-life instrument underwent a rigorous translation process, which involved independent translations, review and discussion of discrepancies, back translation, comparison with the original scales, and pilot-testing with Korean-speaking individuals. The translations were revised as needed based on their feedback. The study requested that participants complete paper-and-pencil research surveys at 2 different points in time—at baseline and week 4. The surveys were conducted at the intervention location (ie, Hanyang Digital Healthcare Center).

#### Depression

As a measurement tool for depression, this study used PHQ-9 [48]. The PHQ-9 is divided into 9 categories of responses to the question, “How often have you suffered from depression-related problems in the past 2 weeks?” The categories are discomfort, depressed feelings, changes in sleep patterns, fatigue, changes in appetite, guilt or worthlessness, poor concentration, restlessness, and suicidal thoughts. High scores calculated by summing the measured scores indicate high degrees of depressive symptoms or high severity of depression.

#### Perceived Stress

The Korean version of the Perceived Stress Scale [49] was used to measure the perceived stress of the participants. The Perceived Stress Scale has 10 items rated on a 5-point Likert scale (1=“not at all” to 5=“very much”), and it is composed of 2 subfactors of positive perception and negative perception according to the way stress is perceived. To measure the perceived stress levels, positive perception factors were reverse scored and summed with negative perception factors to calculate the total score on the scale.

#### Psychological Well-Being

To measure psychological well-being, this study used the WHO-5 Well-Being Index developed by WHO [50], which consists of 5 items. The tool is structured to respond to the following five questions on a 6-point scale ranging from 0=“never” to 5=“always” in the past 2 weeks: (1) “I felt pleasant and happy,” (2) “I was calm and relaxed,” (3) “I was active and energetic,” (4) “I woke up refreshed in the morning after sleeping,” and (5) “My daily life was full of interesting things.” Therefore, possible well-being scores range from 0 to 25, with high scores indicating high levels of psychological well-being.

#### Quality of Life

To measure quality of life, this study used the quality-of-life scale [51]. The tool consists of 4 items, and each item is answered on a 5-point Likert scale ranging from 1=“strongly disagree” to 5=“strongly agree.” High scores indicate high quality-of-life self-ratings. Sample items are “I am satisfied with my life” and “I try to keep developing myself.”

### Mobile Phone–Based Physical Activity Program

The physical activity program used in this study is mobile based and aims to improve physical fitness factors associated with depression. The program features a series of animated and video demonstrations of exercises that involve repetitive movements, designed to engage and challenge various muscle groups. These movements are performed rhythmically and in a coordinated manner to enhance muscular strength and cardiorespiratory endurance. The program’s exercises are repeated several times during each sequence, and 3D animated images display movements designed to enhance coordination and balance by integrating hand and leg movements in different ways. The program’s movements challenge the hands, feet, and trunk and are designed to improve muscular strength and cardiorespiratory endurance.

**Figure 1:**
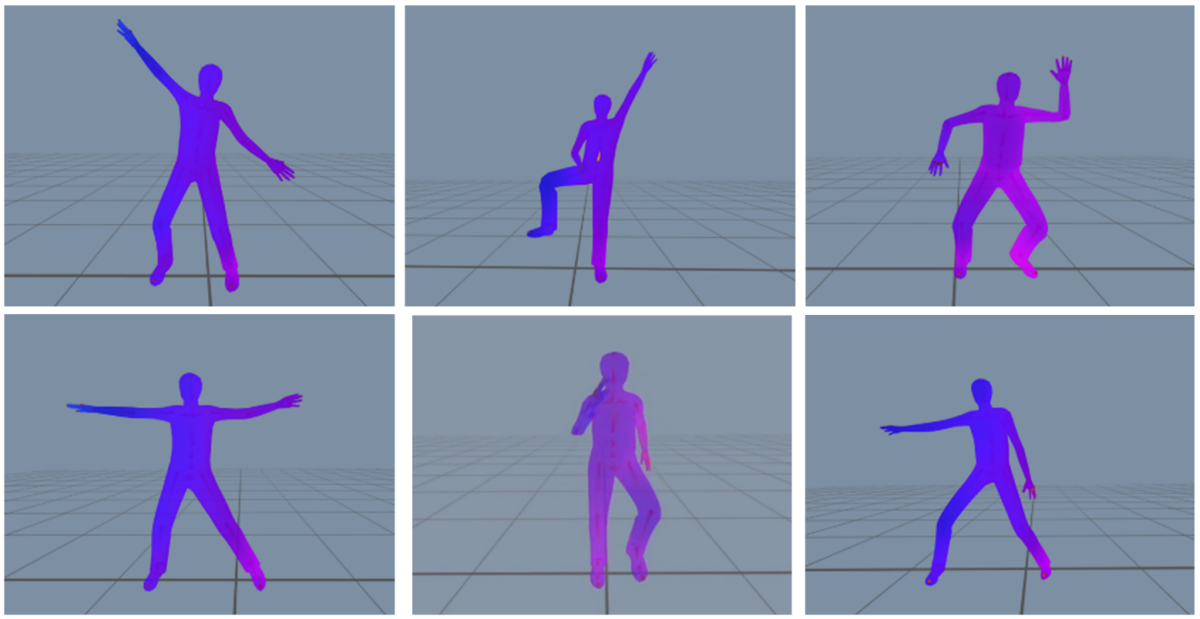
Captured images of the mobile physical activity program’s 3D human character animation.

More specifically, the program is composed of 6 therapeutic movement sequences, each incorporating specific fitness elements (as shown in Figure 1). The first and second sequences were designed to proceed more quickly and include mostly arm exercises. Some specific examples of exercises in these sequences include movements such as alternating arm and leg raises while standing on 1 leg. These exercises require participants to use their core muscles to stabilize their bodies while moving their limbs. In addition, the combination of upper and lower body movements can help to improve cardiovascular fitness by increasing the heart rate and respiratory rate.

The third and fourth sequences in the program focus on coordinated movements of both the arms and legs. The exercises are aimed at expanding the range of motion around the body’s center of gravity and enhancing muscular endurance and balance. It involves standing with the feet hip-width apart and lifting both arms up and out to the sides of the body, reaching toward the sky. The palms of the hands may face each other or be turned outward. The chest and head are lifted upward to create an expansive posture. This movement is often associated with a feeling of openness and energy, as it stretches and opens the chest, shoulders, and arms. By coordinating the movements of both the arms and legs, participants are required to use multiple muscle groups simultaneously, which can help to improve overall body strength and coordination.

The fifth sequence involves walking and running in place to improve cardiorespiratory capacity. This sequence is specifically designed to improve cardiorespiratory capacity or the ability of the body’s cardiovascular and respiratory systems to efficiently deliver oxygen and nutrients to the muscles during physical activity. To perform this sequence, the participant simply walks or runs in place, lifting their feet off the ground and moving their arms back and forth in a natural rhythm. Walking and running in place can be modified to increase the intensity of the exercise by incorporating variations such as high knees, heel kicks, or lateral shuffles. These variations can challenge the cardiovascular and respiratory systems by increasing the intensity and demand of the exercise.

The sixth sequence is designed to enhance coordination between the arms and legs, with the aim of increasing the range of motion around joints. For example, 1 exercise involves lunging forward with 1 leg while simultaneously extending the arm out in front of the body and then alternating sides. Another exercise involves standing with the feet shoulder-width apart and lifting both arms straight up above the head while simultaneously bending the knees and lowering the body into a squat position. Participants may then return to the standing position while lowering the arms to the sides of the body.

In addition, the program’s movement exercises consist of actions with characteristics similar to gestures associated with the expression of positive emotions (joy and happiness). The body motions associated with expressions of joy and happiness have the characteristics of expansiveness, wherein the upper extremities and upper body move upward, with the arms extending laterally [52-55]. This typically involves raising both arms and reaching them outward to the sides of the body while also lifting the chest and head upward to create an expansive, uplifting posture. This upward stretching of the hands also has a stress-reducing effect [56,57]. The participants follow the movements of positive emotional expression while upbeat music plays in the background, which can also reduce depression. Moving in rhythm requires the user to engage in active behavior as opposed to psychomotor retardation, which is a behavioral trait associated with depression.

Only participants in the treatment group were instructed to engage in physical activity while watching videos with physical activity content 5 times a week for 4 weeks. The videos consisted of a total of approximately 20 minutes of physical activity content and were delivered to the participants’ mobile devices through a YouTube link at the same time every day. The researchers did not provide additional treatment or counseling related to the program content.

### Data Analysis

To evaluate the effectiveness of the mobile-based physical activity program for the treatment group and the waitlist group, the self-report questionnaire scores were compared and analyzed using SPSS Statistics for Windows (version 22.0; IBM Corp). To evaluate the impact of the program, we first conducted a 2 (condition) × 2 (time) repeated-measures ANOVA with pretreatment and posttreatment measures and group as independent variables. Following this analysis, we conducted paired-samples 2-tailed *t* tests to compare pretreatment and posttreatment measurements within each group, to gain a more detailed understanding of the data.

## Results

### Recruitment

**Figure 2:**
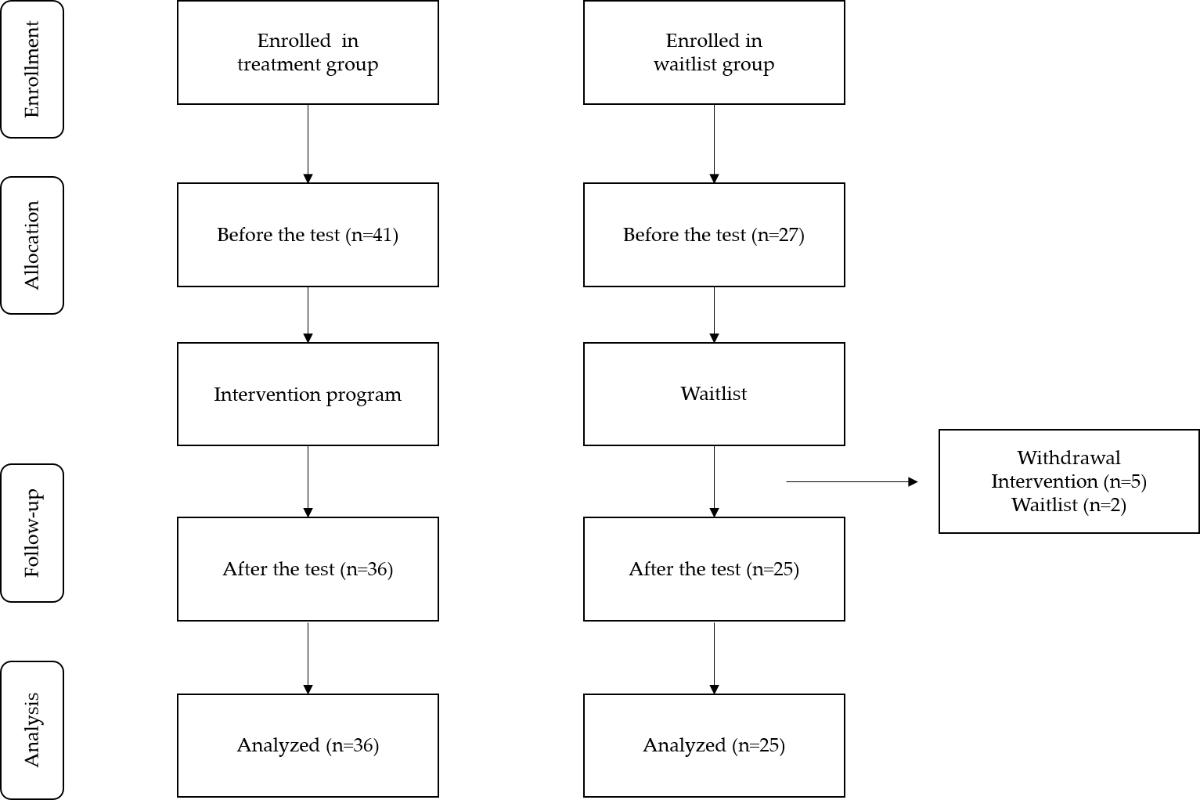
Study flow diagram.

Figure 2 shows the CONSORT (Consolidated Standards of Reporting Trials) diagram, which illustrates the participant flow throughout the study. Of the 68 initial participants, 41 (60%) were assigned to the treatment group and 27 (40%) were assigned to the waitlist group. Overall, 88% (36/41) of the participants completed the follow-up assessment in the treatment group, and 93% (25/27) of the participants completed it in the waitlist group.

The participants of this study were 68 adults aged between 18 and 65 years, who voluntarily expressed their intentions to participate. The selected participants were randomly divided into a treatment group (41/68, 60%) and a waitlist group (27/68, 40%). The treatment and waitlist groups were not balanced in terms of participant numbers because most participants expressed their intention to participate voluntarily on the premise that they would be assigned to the intervention group. Overall, 12% (5/41) of participants in the treatment group and 7% (2/27) of participants in the waitlist group dropped out owing to personal reasons. Thus, a total of 61 participants were included in the final data analysis (Table 1). Specifically, of the total 61 participants, the treatment group consisted of 36 (59%) participants, ranging in age from 20 to 54 years, including 17 (47%) male participants and 19 (53%) female participants. Among these 36 participants, 25 (69%) were unmarried and 11 (31%) were married. Regarding educational background, the 36 participants were distributed as follows: 10 (28%) had completed high school, 10 (28%) were college graduates, and 10 (28%) had graduate degrees, with the lowest number of participants having a high school diploma (n=6, 17%). In terms of occupation, of the 36 participants, 16 (44%) were students, 14 (39%) were regular workers, and 3 (8%) were nonregular workers. The waitlist group consisted of a total of 41% (25/61) participants, ranging in age from 19 to 49 years, including 56% (14/25) male participants and 44% (11/25) female participants. Among the 25 participants, 24 (96%) were unmarried and 1 (4%) was divorced. Regarding educational background, of the 25 participants, the most common level had 13 (52%) participants with a high school diploma, followed by 10 (40%) participants with graduate school or higher education, and 2 (8%) participants with a college degree.

**Table 1:** Baseline participant characteristics.

Characteristics		All participants (N=68), n (%)	Intervention group (n=41), n (%)	Waitlist group (n=27), n (%)
**Sex**				
	Male	33 (49)	19 (46)	14 (52)
	Female	35 (51)	22 (54)	13 (48)
**Marital status**				
	Single	55 (81)	29 (71)	26 (96)
	Married	12 (18)	12 (29)	0 (0)
	Divorce	1 (1)	0 (0)	1 (4)
**Education**				
	High school graduation	6 (9)	6 (15)	0 (0)
	College	27 (40)	12 (29)	15 (56)
	Graduation from university	13 (19)	11 (27)	2 (7)
	Graduate student	22 (32)	12 (29)	10 (37)
**Employment**				
	Student	35 (51)	19 (46)	16 (59)
	Not working	3 (4)	3 (7)	0 (0)
	Full time	19 (28)	16 (39)	3 (9)
	Part time	11 (16)	3 (7)	8 (30)
**Monthly income (KRW ₩ [US $])**				
	No income	15 (23)	7 (17)	8 (30)
	<500,000 (<380.6)	10 (15)	5 (12)	5 (19)
	510,000-1,000,000 (388.22-761.21)	6 (9)	3 (7)	3 (11)
	1,010,000-1,500,000 (768.82-1141.81)	7 (10)	7 (17)	0 (0)
	1,510,000-2,000,000 (1149.43-1522.42)	5 (7)	2 (5)	3 (11)
	2,010,000-2,500,000 (1503.03-1903.02)	7 (10)	2 (5)	5 (19)
	2,510,000-3,000,000 (1910.63-2283.63)	3 (4)	1 (2)	2 (7)
	3,010,000-4,000,000 (2291.24-3044.84)	5 (7)	5 (12)	0 (0)
	>4,000,000 (>3052.45)	10 (15)	9 (22)	1 (3)

### Changes Observed in the Treatment Group and Waitlist Group

First, Tables 2 and 3 summarize the changes (in the treatment and waitlist groups, respectively) in the investigated variables between baseline and after the implementation of the mobile phone–based physical activity program developed for this study. In the treatment group, the mean depression score significantly decreased from 7.08 (SD 5.49) to 4.64 (SD 4.07; *t*_70_=2.14, *P*=.03; Cohen *d*=0.50). The mean depression score of the waitlist group also decreased (from 6.72, SD 5.43 to 5.08, SD 3.4), but this change was not statistically significant (*t*_48_=1.27, *P*=.20; Cohen *d*=0.36). There was significant decrease in the mean perceived stress score of the treatment group (from 2.95, SD 0.51 to 2.72, SD 0.5; *t*_70_=1.99, *P*=.04; Cohen *d*=0.46). In the waitlist group, the mean perceived stress score decreased from 2.82 (SD 0.58) to 2.74 (SD 0.45), but this change was not statistically significant (*t*_48_=0.59, *P*=.55; Cohen *d*=0.15).

The mean psychological well-being score of the treatment group increased from 2.63 (SD 0.80) to 2.96 (SD 0.92) between baseline and after the treatment, but this change was not statistically significant (*t*_70_=−1.63, *P*=.10; Cohen *d*=0.38). In the waitlist group, there was slight increase in the mean psychological well-being score from 2.91 (SD 0.80) to 3.04 (SD 0.62; *t*_48_=−0.63, *P*=.53; Cohen *d*=0.18). Finally, the mean quality-of-life scores increased from 2.89 (SD 0.92) to 3.27 (SD 0.88) and from 3.12 (SD 0.85) to 3.33 (SD 0.79) in the treatment and waitlist groups, respectively, between baseline and after the treatment. However, neither of these increases was statistically significant (*t*_70_*=*−1.76, *P*=.08; Cohen *d*=0.42 for the treatment group; *t*_48_=−0.89, *P*=.37; Cohen *d*=0.25 for the waitlist group).

**Figure 3:**
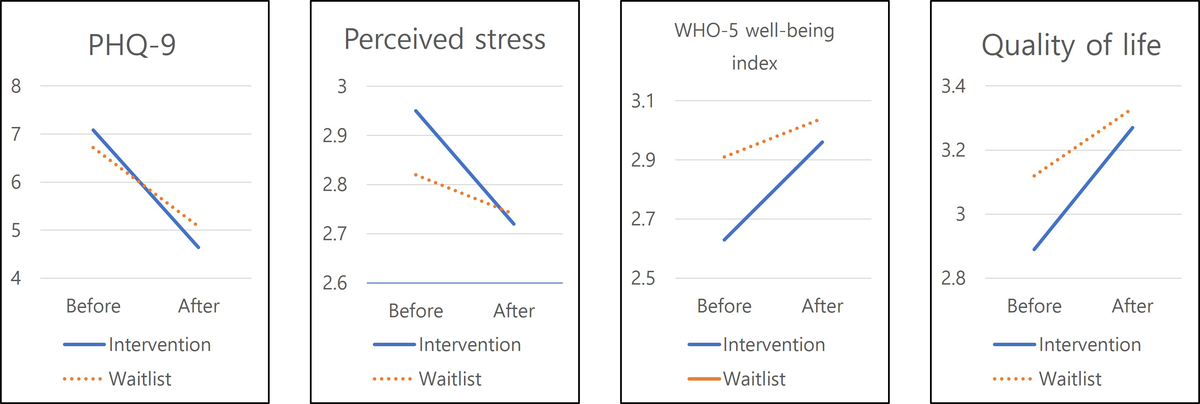
Interaction analysis results. PHQ-9: Patient Health Questionnaire–9; WHO-5: World Health Organization–5.

Figure 3 shows the changes in the treatment and waitlist groups, between baseline and program completion, according to repeated-measures ANOVA of the depression, perceived stress, psychological well-being, and quality-of-life scores. As shown in Table 3, none of the evaluated changes were statistically significant in association with the mobile phone–based exercise. However, accounting for all participants (irrespective of group), depression levels (*F*_1,60_=15.63; *P*=.001; *η*_p_^2^=0.21) decreased significantly after treatment relative to baseline. However, according to the ANOVA, overall, there were no significant changes between baseline and after treatment in perceived stress (*F*_1,60_=7.58; *P*=.25; *η*_p_^2^=0.04), psychological well-being (*F*_1,60_=6.97; *P*=.35; *η*_p_^2^=0.03), or quality of life (*F*_1,60_=3.56; *P*=.06; *η*_p_^2^=0.10).

**Table 2:** Mean differences between pretreatment and posttreatment scores in the treatment group.

	Pretreatment score, mean (SD)	Posttreatment score, mean (SD)	*t* test (*df*)^a^	*P* value	Cohen *d*
Depression	7.08 (5.49)	4.64 (4.07)	2.14 (70)	.03	0.50
Perceived stress	2.95 (0.51)	2.72 (0.49)	1.99 (70)	.04	0.46
Psychological well-being	2.63 (0.80)	2.96 (0.92)	−1.63 (70)	.10	0.38
Quality of life	2.89 (0.92)	3.27 (0.88)	−1.76 (70)	.08	0.42

^a^2-tailed *t* test.

**Table 3:** Mean differences between pretreatment and posttreatment scores in the waitlist group.

	Pretreatment score, mean (SD)	Posttreatment score, mean (SD)	*t* test (*df*)^a^	*P* value	Cohen *d*
Depression	6.72 (5.43)	5.08 (3.4)	1.27 (48)	.20	0.36
Perceived stress	2.82 (0.58)	2.74 (0.45)	0.59 (48)	.55	0.15
Psychological well-being	2.91 (0.80)	3.04 (0.62)	−0.63 (48)	.53	0.18
Quality of life	3.12 (0.85)	3.33 (0.79)	−0.89 (48)	.37	0.25

^a^2-tailed *t* test.

Second, independent-samples 2-tailed *t* tests were performed on the pretreatment results to evaluate the differences between the treatment group and the waitlist group at baseline. Table 4 summarizes the characteristics of the participants in the treatment group and the waitlist group. Regarding the negative well-being indicators, the level of depression was higher in the treatment group (mean 7.08, SD 5.49) than in the waitlist group (mean 6.72, SD 5.43). Perceived stress was also higher in the treatment group (mean 2.95, SD 0.51) than in the waitlist group (mean 2.82, SD 0.58). However, there were no statistically significant differences between the groups in terms of depression (*t*_59_=−0.25, *P*=.79; Cohen *d*=0.06) or perceived stress (*t*_59_=−0.92, *P*=.36; Cohen *d*=0.23). Regarding the pretreatment positive well-being indicators, psychological well-being (mean 2.91, SD 0.80) and quality of life (mean 3.12, SD 0.85) were higher in the waitlist group than in the treatment group (mean 2.63, SD 0.80 for psychological well-being; mean 2.89, SD 0.92 for quality of life), but these differences were not statistically significant (*t*_59_=1.33, *P*=.18; Cohen *d*=0.35 for psychological well-being; *t*_59_=0.96, *P*=.34; Cohen *d*=0.26 for quality of life).

Finally, Table 5 summarize the posttreatment intergroup comparisons. The mean scores of the waitlist group for all variables—depression (mean 5.08, SD 3.45), perceived stress (mean 2.74, SD 0.45), psychological well-being (mean 3.04, SD 0.62), and quality of life (mean 3.33, SD 0.79)—were higher than those of the treatment group (mean 4.64, SD 4.07 for depression; mean 2.72, SD 0.49 for perceived stress; mean 2.96, SD 0.92 for psychological well-being; and mean 3.27, SD 0.88 for quality of life). However, none of the posttreatment intergroup differences were statistically significant (*t*_59_=0.44, *P*=.66; Cohen *d*=0.12 for depression; *t*_59_=0.12, *P*=.90; Cohen *d*=0.04 for perceived stress; *t*_59_=0.34, *P*=.73; Cohen *d*=0.10 for psychological well-being; and *t*_59_=0.26, *P*=.79; Cohen *d*=0.07 for quality of life).

**Table 4:** Pretreatment results of the treatment and waitlist groups.

	Treatment group score, mean (SD)	Waitlist group score, mean (SD)	*t* test (*df*)^a^	*P* value	Cohen *d*
Depression	7.08 (5.49)	6.72 (5.43)	−0.25 (59)	.79	0.06
Perceived stress	2.95 (0.51)	2.82 (0.58)	−0.92 (59)	.36	0.23
Psychological well-being	2.63 (0.80)	2.91 (0.80)	1.33 (59)	.18	0.35
Quality of life	2.89 (0.92)	3.12 (0.85)	0.96 (59)	.34	0.26

^a^2-tailed *t* test.

**Table 5:** Posttreatment results of the treatment and waitlist groups.

	Treatment group score, mean (SD)	Waitlist group score, mean (SD)	*t* test (*df*)^a^	*P* value	Cohen *d*
Depression	4.64 (4.07)	5.08 (3.45)	0.44 (59)	.66	0.12
Perceived stress	2.72 (0.49)	2.74 (0.45)	0.12 (59)	.90	0.04
Psychological well-being	2.96 (0.92)	3.04 (0.62)	0.34 (59)	.73	0.10
Quality of life	3.27 (0.88)	3.33 (0.79)	0.26 (59)	.79	0.07

^a^2-tailed *t* test.

## Discussion

### Summary

Owing to the stigma and cost barriers associated with psychiatric treatment, alternative methods that are easily accessible and user-friendly are needed to improve depression and stress management among the South Korean population. This study sought to investigate the effectiveness of a mobile phone–based physical activity program on depression among adults in South Korea. Scales to evaluate the severity of depressive symptoms, perceived stress, psychological well-being, and quality of life were assessed twice (before and after program use) after allocating the adults who participated in the study to a treatment group and a waitlist group. The analysis uncovered disparities in the impact of time on depression between the treatment group and the waitlist group. In addition, the pretreatment and posttreatment comparisons indicated decreased depression and perceived stress scores in the treatment group. The study suggests that digital physical activity applications can be a valuable tool in promoting mental health and well-being, particularly in populations with limited access to traditional mental health services. These findings are particularly relevant in the context of the COVID-19 pandemic, where stress and depression have become more prevalent.

### Principal Findings

The strength of this study lies in its demonstration of the effectiveness of a mobile phone–based physical activity program in mitigating depressive symptoms and other mental health indicators among adults. This study adds to the growing body of research exploring the potential of digital applications for depression treatment, which is actively being developed and verified internationally. In recent years, digital physical activity applications have gained popularity, with fitness tracking apps such as MyFitnessPal, Fitbit, and Strava being actively developed. These apps use sensors to track users’ physical activity, including steps taken, distance traveled, and calories burned. They also allow users to set goals and track progress over time. Virtual reality fitness games, such as RingFit, Beat Saber, and BoxVR, are another area of ongoing development in digital physical activity applications. These games use virtual reality technology to create immersive environments that users can interact with through physical activity. The gamification of physical activity is also an area of ongoing development through apps such as Pokemon Go and Zombies, Run! This study is particularly important, as it demonstrates the effectiveness of a home-based physical activity program for reducing depressive symptoms and perceived stress among adults in South Korea. The cost-effectiveness of this treatment program adds to its potential as a means of promoting mental health and physical activity in populations with limited access to traditional mental health services. Therefore, this study provides an important foundation for further studies into the efficacy of digital physical activity programs as a means of promoting mental health and physical activity, particularly in populations experiencing high levels of stress and depression.

Recently, especially since the onset of the COVID-19 pandemic, many studies have been conducted to investigate the effectiveness of mobile phone–based health apps in promoting physical activity, and it is evident that such apps are promising tools for promoting physical activity [58]. The mobile phone–based physical activity program evaluated in this study was shown to be effective; thus, our results concur with previous evidence showing that physical activity is associated with improvements in depression and perceived stress. Our mobile phone–based exercise program facilitated a combination of muscular strength training and aerobic training, which has been previously shown to be effective. Although there is robust evidence for the beneficial effects of exercise on mental health, various studies have supported different types and modes of exercise; for example, 16 weeks of aerobic exercise (dance, jump, and traditional games) improved the depressive symptoms of adults with depression [59], and 8 weeks of high-intensity strength training (at 80% of the 1-repetition maximum) was beneficial in the treatment of older adults with depression [60]. However, regardless of the type and mode of exercise, physical activity is beneficial for mental health. Therefore, any type of exercise using mobile phone–based physical activity is recommended for mental health.

Regular physical activity and exercise are effective therapies for most chronic diseases, including mental disorders [15,61]. For example, a recent meta-analysis by Pearce et al [24], which included 15 studies and 2,110,588 person-years, demonstrated that participants accumulating half the recommended amount of physical activity had an 18% lower risk of depression than adults without physical activity. Furthermore, adults accumulating the recommended volume of 8.8 marginal metabolic equivalent task hours per week had a 25% lower risk of diminishing potential benefits. Several systematic reviews have found that exercise can reduce the symptoms of depression with a moderate to large effect size and can be a useful addition to pharmacotherapy and psychotherapy [25-30]. Exercise has been shown to counteract reductions in the secretion of neurotransmitters, such as dopamine and serotonin, thus having a positive effect on emotions and reducing the severity of psychological symptoms, such as anxiety and depression. He et al [62] reported that 10 weeks of voluntary running exercise significantly increased serotonin, dopamine, and norepinephrine levels in the hippocampus, which had been reduced in rats with chronic mild stress.

The results of this study indicate that the mobile phone–based physical activity program developed for this study led to a significant decrease in perceived stress among participants in the treatment group, whereas the waitlist group showed no significant change. The significant reduction in perceived stress observed in the treatment group suggests that mobile phone–based physical activity programs could be a viable option for individuals seeking to reduce stress levels [63]. There are several potential underlying mechanisms that may explain the significant effect of the mobile phone–based physical activity program on perceived stress. First, physical activity has been shown to stimulate the release of endorphins, which are natural chemicals that can improve mood and reduce feelings of stress and anxiety. These endorphins can counteract the negative feelings associated with stress and promote a sense of well-being. Second, engaging in physical activity may help individuals to divert their mind from stressful thoughts or situations, providing a temporary escape and reducing overall levels of perceived stress. In other words, when individuals engage in physical activity, they may shift their attention away from the stressors or negative thoughts that are causing stress. This can provide a temporary break from stress, allowing the individual to clear their mind and return to their stressors with a renewed focus and sense of calmness. The theoretical implications of this study are also important. According to the stress and coping theory [64], stress is a result of the relationship between an individual and their environment and the way in which they perceive and cope with stressors. This theory suggests that stress can be managed through the development of effective coping strategies, such as engaging in physical activity. The findings of this study support the stress and coping theory, as the mobile phone–based physical activity program was found to be effective in reducing perceived stress levels among participants. Furthermore, the results of this study provide support for the use of digital interventions in mental health treatment. The use of mobile phone–based physical activity programs is an example of how technology can be leveraged to improve mental health outcomes. This study adds to the growing body of research on the effectiveness of digital interventions for mental health [65,66]. The use of mobile phone–based physical activity programs could be a useful addition to the mental health treatment landscape, particularly in populations with limited access to traditional mental health services.

On the basis of the results of this study, it appears that the mobile phone–based physical activity program did not have significant effect on perceived stress, psychological well-being, or quality of life among participants. Although there was slight increase in the mean scores for psychological well-being and quality of life in both the treatment and waitlist groups, these increases were not statistically significant. It is possible that the lack of significant findings in this study could be owing to various factors. For example, the duration and frequency of the program may not have been sufficient to produce significant changes in mental health outcomes. In addition, individual differences in adherence to the program and motivation to engage in physical activity could have influenced the results.

Despite the lack of significant findings, the use of mobile phone–based physical activity programs as a cost-effective and accessible alternative for the treatment of depression and perceived stress among adults should not be dismissed [67]. It is important to continue exploring and refining such programs to maximize their potential benefits for mental health. Moreover, the nonsignificant changes in quality of life and psychological well-being in the waitlist group suggest that simply being enrolled in the program could have a positive effect on these outcomes. Future studies should explore the potential placebo effect of simply being enrolled in a program and the potential benefits of combining mobile phone–based physical activity programs with other interventions such as cognitive behavioral therapy or medication.

### Practical Implications

On the basis of the findings of this study, practitioners can suggest mobile phone–based physical activity programs as a cost-effective and accessible alternative for the treatment of depression and perceived stress among adults. These programs can be easily implemented at home and can help individuals overcome time and accessibility barriers associated with physical activity.

For example, a practitioner working in a mental health clinic can recommend a mobile phone–based physical activity program to their patients who are experiencing symptoms of depression or perceived stress. The program can be used as a complement to their existing treatment or as a stand-alone intervention. The practitioner can provide guidance about how to use the program, monitor the patient’s progress, and adjust the treatment plan accordingly. The expected outcomes of using such a program can be a significant decrease in depressive symptoms and perceived stress levels, leading to improved mental and physical well-being. Patients may also experience increase in motivation, self-efficacy, and overall quality of life. However, it is important to note that the program may not have a significant effect on other variables such as psychological well-being or quality of life, as observed in this study.

Furthermore, a community center or gym can offer a mobile phone–based physical activity program to members who are unable to attend in-person exercise classes. This can help make physical activity more accessible to individuals who may have difficulty in traveling to a gym or attending a class during scheduled times. In addition, the program can be offered at a lower cost than that of in-person classes, making it a more affordable option for those with a tight budget.

Finally, an employer can provide a mobile phone–based physical activity program to their employees as part of a workplace wellness program. This can help employees manage stress and improve their mental health, which could lead to increased productivity and job satisfaction. In addition, offering such a program can demonstrate that the employer values employee well-being and is committed to promoting a healthy work environment.

### Limitations and Future Research Directions

The limitations of this study were as follows. First, the treatment period of 4 weeks was short, and it limited the confirmation of the prevention effect over time. Therefore, more rigorous evaluations of the effectiveness of such programs—via randomized controlled trials in which participants are randomly assigned to treatment and waitlist groups—should be conducted, and such trials should be conducted over sufficient treatment periods of at least 3 months.

Second, the participants of this study consisted of members of the general public with low levels of depressive symptoms. Thus, it cannot be generalized that findings apply to adults with high levels of depressive symptoms. Although it is worthwhile to investigate the effectiveness of digital applications for this type of sample in an attempt to prevent severe depression, the program may need to target patients who have mild to severe depressive symptoms for treatment purposes.

Third, although the mobile phone–based physical activity program was modified and supplemented through preliminary studies, many factors need to be corrected in the course of the implementation of the program. Moreover, considering the feedback of the participants who completed 4 weeks of the program, it is possible that the lack of graphical detail in the content composition and errors in the program acted as factors that lowered the motivation of the participants. Therefore, there is a need to improve participation levels by further supplementing and improving the treatment program based on the feedback of the study participants.

Finally, although this study did not find significant changes in psychological well-being or quality of life among participants, the potential benefits of mobile phone–based physical activity programs for mental health should not be overlooked. Further studies are needed to identify optimal program characteristics and implementation strategies to maximize their efficacy in improving psychological well-being and quality of life.

### Conclusions

In conclusion, this study investigated the effects of mobile phone–based physical activity programs on depression, perceived stress, psychological well-being, and quality of life. The findings indicated that 4 weeks of training using the program significantly reduced the participants’ levels of depression and perceived stress. This study suggests that practitioners can recommend mobile phone–based physical activity programs as a cost-effective and accessible alternative for treating depression and perceived stress among adults. Practitioners can offer guidance to patients about how to use the program and monitor their progress while adjusting their treatment plan accordingly. This program can be used as a complement to existing treatment or as a stand-alone intervention. Expected outcomes may include significant reduction in depressive symptoms and perceived stress levels, increased motivation and self-efficacy, and improved overall quality of life. However, practitioners should be aware that the program may not have significant effect on other variables such as psychological well-being or quality of life. Community centers or gyms can also offer such programs to members who are unable to attend in-person classes, making physical activity more accessible and affordable. Employers can also provide these programs as part of a workplace wellness program, thus promoting a healthy work environment and improving employee productivity and job satisfaction.

## Appendix

Multimedia Appendix 1 CONSORT-eHEALTH checklist (V 1.6.1).

## Abbreviations

CONSORT: Consolidated Standards of Reporting Trials
PHQ-9: Patient Health Questionnaire–9
WHO: World Health Organization
